# Perceptions of community health workers on teenage pregnancy in rural Limpopo: A qualitative study

**DOI:** 10.4102/phcfm.v16i1.4296

**Published:** 2024-03-24

**Authors:** Rakgadi G. Malapela, Sheillah H. Mboweni, Patrone R. Risenga

**Affiliations:** 1 Department of Health Studies, School of Social Sciences, University of South Africa, Pretoria, South Africa

**Keywords:** community health workers, exploratory, perceptions, qualitative study, teenage pregnancy

## Abstract

**Background:**

Despite measures put in place to combat teenage pregnancy, the rate remains high. Community health workers (CHWs) are a cadre of health workers that can help put measures in place to reduce teenage pregnancy in the communities in which they live and work.

**Aim:**

This article aims to gain a deeper understanding of CHWs’ perceptions regarding teenage pregnancy in the rural districts of Limpopo province.

**Methods:**

An exploratory qualitative study approach was employed to collect data from CHWs in two rural districts of Limpopo. A non-probability purposive sampling approach was used to choose 81 CHWs. Eight focus group discussions (FGDs) were organised, and audio recorded to collect data from participants. The discussions were 2–3 h long and conducted in English, and data saturation was attained by the fifth FGDs.

**Results:**

An eight-step tech’s content analysis approach was employed to deductively code, analyse and summarise data into themes. Three themes emerged: the prevalence of teenage pregnancy in rural villages, factors contributing to teenage pregnancy and challenges faced by CHWs when dealing with teenage pregnancy.

**Conclusion:**

The study’s findings revealed that CHWs face challenges in their communities when offering appropriate teen pregnancy services and CHWs believe that teen pregnancy numbers remain high. There is a significant barrier in combating teenage pregnancy; if contraceptives are not acceptable to the community, the only solution and option for combating teenage pregnancy is abstinence.

**Contribution:**

The CHWs presented their insights of teenage pregnancy in rural communities. The outcomes of this study could help clinical practise, schools, communities, youth-friendly services, policymakers and other non-governmental organisations reduce teenage pregnancy.

## Introduction

The rate of adolescent pregnancy remains startling and concerning. Special consideration must be given to solutions needed to overcome this issue. According to Chemutai et al., any pregnancy occurring between the ages of 10 and 19 is classified as a teenage pregnancy.^1,2^ Each year, it is estimated that 2 million girls under the age of 15, and 21 million between the ages of 15 and 19 become pregnant. Furthermore, around 16 million of these girls give birth every year, with 90% of them living in low-income countries, including South Africa.^1^ According to a South African study, teenage pregnancy is increasing year by year in all provinces, with the rural provinces being more affected than their urban counterparts.^3^ Limpopo, Mpumalanga and the Eastern Cape have the highest rates of teenage pregnancies among the rural provinces, whereas Gauteng and the Western Cape have the highest rates among the urban provinces (NDoH 2020).^4^

According to a global average study of adolescents aged 15–19, sub-Saharan Africa has 99 births per 1000 teenagers, Western Europe 8 births per 1000, and South Africa 41 births per 1000 adolescents.^5^ This translates to almost 14% of South African teenagers under the age of 19 falling pregnant. Similarly, it has been reported that in South Africa, over 16 million girls aged 15–19, including one million under the age of 15, are giving birth, which is recognised as a severe public health issue.^6,7,8^ Furthermore, the number of deliveries among South African girls aged 10–14 is said to have climbed by 48.7% from 2726 in 2017/2018 to 4053 in 2020/2021.^3^

The United Nations (2015) Sustainable Development Goal 3 (targets 3.1 and 3.7) prioritises the need to reduce maternal and child mortality rates.^9^ Teenage pregnancy has a negative impact on the well-being of the youth as it contributes to high rates of maternal and child mortality and morbidity because of late-term miscarriages, hypertensive problems during pregnancy, haemorrhagic syndromes and premature membrane rupture.^2,8^ Furthermore, babies born to teenage mothers often reflect increased low birth weight rates, preterm and neonatal mortality because of catastrophic consequences. To reduce teenage pregnancy in South Africa, the Department of Basic Education, the Department of Health, and other partners implemented comprehensive sexuality education.^10^ This was done to improve adolescent sexual and reproductive health education.

Research conducted in selected South African districts established the fact that adolescent pregnancy is no longer considered a societal challenge; rather, it has become a fashionable and common norm.^11^ Therefore, it is not unexpected that teenage pregnancy rates continue to escalate despite efforts to reduce it, such as the school being mandated to report teenage pregnancy to police.^10^

Community health workers (CHWs) are considered lay health workers and paraprofessionals who have a greater understanding of the community’s culture and language.^12^ According to the World Health Organization (WHO), CHWs are healthcare personnel who have obtained informal training and education and live in the community they serve to improve the performance and results of the health system WHO.^13^ They can provide community-based care and mobilise communities to solve health challenges. Because they receive family planning in-service training, they can impart contraceptive and health education, in addition to conducting home visits and community outreach.^12^ The WHO has advised non-governmental organisations (NGOs) to take rapid action to address the global teenage pregnancy problem.^14^

Existing literature indicates a need for studies that consider community members’ perceptions of teen pregnancy prevention.^11,13,14^ To address this gap, this research project was conducted in Limpopo’s rural areas to determine the CHWs’ perceptions of adolescent pregnancy. The study’s grand tour question is, ‘What are the CHWs’ perceptions of teenage pregnancy?’ The aim of this study is to gain a deeper understanding of CHWs’ perceptions of teenage pregnancy in the rural areas of Limpopo province. It is critical to conduct and participate in research to acquire a better understanding of CHWs’ perceptions about teenage pregnancy. These reinforce the necessity for research into the prevention of teenage pregnancy.^9^

## Research methods and design

### Study design

An exploratory qualitative methodology was used to delve into the experiences, attitudes and beliefs of CHWs in a comprehensive manner, allowing for a rich exploration of the topic. The focus group discussions (FGDs) were conducted to collect data. The purpose of this study was to gain a deeper understanding of CHWs’ perceptions of teenage pregnancy in the rural areas of Limpopo province. The research objectives of this study were threefold: to explore the perceptions of CHWs regarding teenage pregnancy, to determine the contributing factors to teenage pregnancy, and to describe the challenges faced by CHWs when dealing with teenage pregnancy.

### Setting

The research was conducted in two rural regions of South Africa’s Limpopo province, namely Mopani and Vhembe. Limpopo is in the far north of South Africa and shares borders with three adjacent countries: Botswana, Zimbabwe and Mozambique. It is primarily a rural province that ranks second in South Africa in terms of teenage pregnancy. Mopani has five municipalities, 354 villages, and 125 wards, and Vhembe has four municipalities, 898 villages, and 127 wards.^15,16^

### Study population and sampling strategies

Participants CHWs were recruited from NGOs based in the rural Limpopo districts of Mopani and Vhembe. The CHWs were aged between 20 and 79 years and have been providing community and home-based care services, including dealing with the challenges affecting adolescents and the youth in their local community or village for more than a year with the support and supervision of retired nurses. A non-probability purposive sampling strategy was adopted. The recruitment process lasted from March through June 2022. The data were collected from July 2022 to October 2022. Eighty-one CHWs participated in the study. In each district, four focus groups were held. By the fifth FGD data saturation was achieved.

### Data collection

Eight focus groups were held, each containing 8–9 participants, giving a final total of 81 participants in the study. The appropriate number of FGD ranges between 6 and 10 depending on the size of the groups.^17^ Each discussion lasted about 2–3 h per group. A discussion guide was utilised to facilitate the discussion process, which was then followed by probing questions. The entire process took place in the community’s conference venue. Guidance was utilised to ensure that the debate ran smoothly and consistently. Participants were asked open-ended questions that described their perspectives, experiences, points of view and attitudes towards teenage pregnancy. For improved comprehension, questions were prepared in English and further explained in their native tongue, Xitsonga, Sepedi and Tshivenda. The conversations were audio recorded with the permission of the participants.

To assess the relevance and quality of the questions, the discussion guide was pilot tested with five participants from a district not included in the study. The question of ‘what is the prevalence of teenage pregnancy in your area’ was seen as being complex. Following pilot testing, the questions were changed and simplified as needed. This question then became ‘what is the rate of adolescent pregnancy in your area?’ The results of the five pilot study participants were not included in the study’s findings.

### Data analysis

Tesch’s eight steps content analysis was used to methodically analyse textual material and to interpret the data into themes. The transcripts were then examined by the researchers and existing data were coded. The data from the focus groups were used to construct themes, categories and sub-categories. The steps followed are: (1) To ensure that everything was captured, the researchers studied the transcribed data, and wrote down thoughts as they presented themselves. (2) The meaning of all transcriptions was checked. (3) The data were organised into clusters with similar themes grouped together. (4) Formulated subjects were assigned codes that were written adjacent to relevant text parts. (5) All three researchers performed verification to ensure that no new categories or codes emerged. All three researchers have prior experience with qualitative research, and disagreements were aired and resolved. (6) Based on the study findings, categories were defined and developed. (7) A final judgement was made for each category, and the codes were written logically. The researchers accomplished this through rigorous evaluation and conversations. (8) Data material from each category was gathered in one location and a preliminary analysis was performed. Existing data were recorded and logically displayed.^18^

To assure the study’s trustworthiness, credibility, transferability, reliability and conformability were improved throughout the study. An extra hour or two was spent with the participants and their comments documented to enable correct interpretation while extracted quotes increased credibility. A detailed description of the participants’ information on the phenomenon under research ensured transferability. Dependability was improved by describing and comparing field notes to FGD audio recordings. A member check was performed to ensure that what the researchers recorded was correct and a real reflection of participant opinions. The three researchers ensured conformability by scrutinising the data-collection procedure, assessing the data, and validating the interpretations and themes derived from the study findings. Adding verbal quotes gathered during the sessions increased authenticity.^18,19^

### Ethical considerations

The study was conducted in compliance with the Helsinki Declaration, as revised in 2013, to promote and protect participants’ health and rights, even if they had volunteered to participate. The UNISA College of Human Sciences Ethical clearance to conduct this study was obtained from the University of South Africa College of Human Sciences Research Ethics Review Committee. (No. HSHDC/517/2016), with an approved period of 29 April 2021 to 29 April 2024. To protect the participants, ethical considerations were followed. The study’s participants were all above the age of 18, and their participation was entirely voluntary. Those who agreed to participate were asked to complete written consent forms. Freedom to withdraw from the study at any time was indicated. Numbers were used instead of participant names, to maintain confidentiality and anonymity. The participants were requested not to discuss the information presented with others who were not involved in the study. During the FGDs, the researchers used bracketing to eliminate bias and preconceived conceptions by laying aside presumptions, prejudices, assumptions or prior experiences to observe and document the phenomenon under study.^18^

## Findings

This study included 81 participants ranging in age from 20 to 79 years. The majority were females who had completed their Grade 12 certificates. A bachelor’s degree was the highest qualification. The demographic characteristics of CHWs are shown in Table 1.

**TABLE 1:** Demographic profile of participants (*N* = 81).

Criterion	Frequency	Percentage
**Age (years)**		
20–29	19	23
30–39	38	47
40–49	18	22
50–59	3	5
60–69	2	2
70–79	1	1
**Education**		
High school	23	28
Grade 12	39	48
Certificate	13	16
National Diploma	3	5
Bachelor’s degree	2	2
**Gender**		
Female	72	89
Male	9	11
**Years’ working with community**		
1–2	65	80
3–4	13	16
5–6	3	4
**District**		
Mopani	51	63
Vhembe	30	37

### Perceptions of community health workers who deal with teenage pregnancy in rural districts of Limpopo province

The study findings revealed three themes with matching sub-themes. The themes are the prevalence of teenage pregnancy in rural areas, the factor contributing to teenage pregnancy in rural villages, and the challenges faced by CHWs when dealing with teenage pregnancy. Table 2 outlines the study findings’ themes and sub-themes.

**TABLE 2:** Themes and sub-themes of the study findings.

Themes	Sub-themes
The prevalence of teenage pregnancy	Perceived high number of teenage pregnancies
Factors contributing to teenage pregnancy	Lack of knowledge and awareness about sexual and reproductive health Minimal access to healthcare services Miscommunication in churches Poverty and lack of financial support Poor parenting style Peer pressure Disrupted and disorganised families. Social media and technology Alcohol and substance abuse Lack of recreational facilities Lack of a sense of belonging and feelings of not being loved
Challenges faced by CHWs when dealing with teenage pregnancy	Lack of support from parents in the village Lack of respect from teenagers

CHW, community health workers.

### The prevalence of teenage pregnancies in rural villages

The participants stated that teenage pregnancy is highly prevalent in their localities. During the focus groups, almost all participants felt that there is a rise in the number of teenage pregnancy.

### Perceived high number of teenage pregnancies in rural villages

The prevalence of teenage pregnancy is increasing in rural communities, and this is seen as a major concern that must be addressed decisively. Participants reported that, despite measures put in place to reduce teenage pregnancy, there are still high numbers of teenage pregnancies in the rural villages. One participant was recorded shaking their head and saying:

‘I can estimate teenage pregnancy in my area as at 90%.’ (FGDs 1: P3, Female, 35 years old with grade 12 and 2 years of work experience)

### Factors contributing to teenage pregnancies

Participants cited the various factors that they believe contribute to teenage pregnancy in their rural villages that include a lack of knowledge and awareness regarding sexual and reproductive health; a lack of healthcare services; miscommunication in churches; poverty or a lack of financial support; poor parenting style; peer pressure; disrupted and disorganised families; social media and technology; alcohol and substance abuse; a lack of recreational facilities; and a lack of a sense of belonging and feelings of not being loved.

### Lack of knowledge and awareness regarding sexual and reproductive health

Participants expressed their belief that teenagers lack information and awareness about sexual reproductive health. Participants stated that adolescents are not taught about sexuality in their local schools. The following quotes were captured:

‘I was in high school and when we have to learn about sexuality education, we are told to go out for sports instead of learning about it, it seems teachers are afraid to educate us about it.’ (FGDs 1, P1, Female, 25 years old with certificate and 2 years of work experience)

‘Teachers are afraid to teach, most learners do not behave, they became uncontrollable. Laughing when they start showing pictures related to female and male reproductive organs, even us when we visit schools.’ (FGDs 2, P5, Male, 40 years old with diploma and 2 years of work experience)

Other participants added:

‘I only learn more about sexuality education and reproductive health now, when I join the NGO as a CHW, I think I would have also prevented my teenage pregnancy if I knew about it.’ (FGDs 4, P7, Female, 38 years old with certificate and 2 years of work experience)

‘We have never had an awareness [*programme*] where teenage pregnancy and contraceptives were educated, we mostly talk about HIV [*human immunodeficiency virus*] and AIDS [*acquired immunodeficiency syndrome*], sexually transmitted illnesses [*STIs*], tuberculosis [*TB*] and non-communicable diseases [*NCDs*].’ (FGDs 2, P8, Female, 23 years old with certificate and 2 year of work experience)

Participants stated that students are poorly informed about sexual-education in school. Teachers avoid discussing this subject.

### Minimal access to healthcare services

Participants are deeply concerned that most rural villages have limited access to clinics and mobile teams. The following statements supported this view:

‘Most of our villages have no clinics, and [*the*] mobile [*clinic*] does not frequently visit our areas.’ (FGDs 3, P5, Female, 70 years old with diploma and 4 years of work experience)

‘We once had a meeting with chief, to assist us with writing a letter to [*the*] municipality and department of health to have a clinic but with no response.’ (FGDs 3, P4, Female, 60 years old with bachelor and 6 years of work experience)

Others indicated with pain that:

‘We sometimes do not have money and walk by foot to the clinic which is more than 25 kms from our village.’ (FGDs 1, P8, Female, 45 years old with certificate and 3 years of work experience)

‘Imagine a school child who has to go to the clinic after school for contraceptives, it is not possible as the clinic will be closed by time they get there.’ (FGDs 3, P6, Female, 52 years old with certificate 12 and 5 years of work experience)

Most participants mentioned that access to contraception is limited. In other cases, there are no clinic facilities where contraceptives can be obtained.

### Miscommunication in churches

Churches and religion are not helping to minimise or prevent teenage pregnancy because it is anticipated that no sex will occur before marriage, and some religions consider the use of contraception to be taboo and against God’s will. This was captured as follows:

‘The church is not helping much because they discourage the use of contraceptives and sex, however, teenagers are engaging in unprotected sex and fall pregnant while they are Christian.’ (FGDs 1, P7, Female, 30 years old with certificate and 4 years of work experience)

Another angrily expressed that:

‘When the teenage girl fell pregnant, she is disciplined, cut off church activities while the boy continues enjoying and even impregnated other girls in the same church and nothing is being done.’ (FGDs 7, P2, Female, 45 years old with bachelor and 5 years of work experience)

While one added:

‘It is taboo to talk about sex in church, all they say is no sex before marriage and [*it*] is not helping us with this new generation.’ (FGDs 1, P5, Female, 52 years old with certificate and 4 years of work experience)

### Poor parenting style

Poor parenting style is a key issue that contributes to teenage pregnancy in rural villages. Participants stated that:

‘Parents do not talk to their kids while they are in puberty about sex as it is taboo in our culture and most teenagers fell pregnant not knowing how to prevent it.’ (FGDs 7, P6, Female, 37 years old with grade 12 and 2 years of work experience)

Others added:

‘Some parents let their children do as they wish, no discipline, they drink alcohol and can stay away from home for days, they do not care.’ (FGDs 3, P3, Female, 55 years old with diploma and 4 years of work experience)

Participants cited inadequate parenting abilities and a lack of child supervision as contributing causes to teen pregnancy.

### Poverty and lack of financial support

Participants were recorded saying:

‘Yhoo! it is painful. In my village a mother sells her daughter to a man so that they can get money, the child was still young, abused sexually until she fell pregnant at the age of 12 years. That’s where it was discovered.’ (FGDs 2, P1, Female, 35 years old with certificate and 2 years of work experience)

‘Young girls themselves go to taverns and flirt with older men with money, have unprotected sex and end up pregnant.’ (FGDS 2, P9, Male, 25 years old with certificate and 2 years of work experience)

‘Child grant is motivating young girls to have kids so that they can get more money and exposes them the STI and HIV and AIDS as they had unprotected sex.’ (FGDs 8, P8, Female, 35 years old with diploma and 6 years of work experience)

‘Some parents neglect their children, work away from home without anyone to supervise the children, some are alcoholics and do not care about their children.’ (FGDs 3, P7, Female, 33 years old with grade 12 and 2 years of work experience)

Another participant expressed with anger:

‘Some parents encourage their teenagers to fell pregnant in order to get a child grant or trap a man who has money to deal with poverty.’ (FGDs 4: P6, Female, 37 years old with grade 12 and 1 year of work experience)

Participants indicated that poverty is a severe issue in remote villages, and people struggle to make a livelihood by any means possible, which sometimes includes indulging in unlawful or dangerous activities. Teenagers and their parents both engage in dangerous behaviours to obtain financial support from men in higher financial status, which results in teenage pregnancy and/or sexual abuse.

### Disrupted and disorganised families

This was supported by this quote:

‘A mother had a boyfriend who abused her daughter when she is not there and now the child is pregnant.’ (FGDs 1, P2, Male, 35 years old with certificate and 2 years of work experience)

Most families in these rural communities’ villages, according to participants, are disorganised and no longer adhere to indigenous traditional values, family value systems or ubuntu. Some mothers invite boyfriends into their homes, exposing their children to sexual abuse, ultimately resulting in teenage pregnancy.

### Social media and technology

Participants mentioned social media and technology as contributing causes, which was corroborated by the following citations:

‘Social media is a problem, as I also do not know how to block my child’s phone not too access porn and [this] is similar problem with parents in our village as they are not educated or not having knowledge of today technology.’ (FGDs 3, P6, Female, 34 years old with certificate and 2 years of work experience)

‘Teenagers share videos of porn, and they later start to experiment it without knowledge of [*the*] dangers of such, as they have unprotected sex and fell pregnant.’ (FGDs 5, P1, Female, 39 years old with certificate and 1 year of work experience)

Adolescents frequently imitate the sexual activities they observe on social media and the internet further exposes them to pregnancy.

### Alcohol and substance abuse

Participants disclosed that …

‘Teenage girls abuse alcohol and take drugs. When they are drunk or high, they are taken advantage [*of and*] raped, and they do not even know who is responsible for the pregnancy. While someone engages willingly under the influence of alcohol or drugs.’ (FGDs 5, P3, Female, 44 years old with certificate and 2 years of work experience)

Substance addiction, according to participants, is fuelling the rising rate of teenage pregnancy, as many of the youth misuse and abuse alcohol and drugs. This has an impact on their decision-making abilities and exposes them to risky activity.

### Lack of recreational facilities

Participants highlighted that:

‘There are no playgrounds for kids and teenagers to play different sports in our villages.’ (FGDs 8, P1, Female, 58 years old with grade 10 and 4 years of work experience)

‘Even at schools, sports are no longer a priority, school children just go home when it is time for sports.’ (FGDs 1, P3, Female, 32 years old with certificate and 2 years of work experience)

Rural villages lack recreational facilities, thus teenagers in the community are not engaged in sporting activities and resort to substance abuse out of boredom.

### Lack of a sense of belonging and feelings of not being loved

Participants were stating that:

‘Young girls want fancy things and if their parents do not afford, they feel like they are not loved or do not belong and resort in risky relationships.’ (FGDs 3, P4, Male, 30 years old with grade 12 and 2 years of work experience)

‘Some girls just want to prove that they can be loved and engage in promiscuous relationships, ending up pregnant.’ (FGDs 6, P4, Female, 37 years old with certificate and 3 years of work experience)

Participants discovered during household visits and interactions with youngsters in the homes that most teenagers lack a sense of belonging and believe that their parents do not love them. Teenage girls are more likely to seek affection from teenage or older men.

### Peer pressure

Participants reported the following peer pressure quotes:

‘Young girls imitate other girls without knowledge of protected sex or contraceptives and end up pregnant.’ (FGDs 1, P4, Male, 36 years old with certificate and 2 years of work experience)

‘Some young girls envy their peers, that are getting child grant and buying hair styles and clothes, and they decided to deliberately fall pregnant.’ (FGDs 5, P4, Female, 31 years old with grade 12 and 1 year of work experience)

Participants reported that many of the youngsters are subject to peer pressure, and when they fail to resist these pressures, it results in unsafe relationships and engaging in risky sexual activities, which can result in teenage pregnancy.

### Challenges faced by community health workers when dealing with teenage pregnancy

Participants discussed the challenges they face in the field, such as a lack of support from parents in the village and a lack of respect from teenagers in the community, when implementing community awareness campaigns with parents and teenagers to discuss teenage pregnancy and its implications for teenagers’ health, psychosocial, and economic status, as well as the wider community.

### Lack of support from parents in the village

Furthermore, some parents are unaware of the difficulties that teenage mothers confront. These participant statements back up these feelings:

‘When mobilising parents to have a meeting with them, we were scolded and reminded of our childhood and adolescence as some of us also experienced teenage pregnancy, but we have learned our lessons and do not want others to experience the same pain.’ (FGDs 2, P2, Male, 44 years old with grade 10 and 2 years of work experience)

‘Parents threatened us, that we are teaching their kids contraceptives and if they became infertile, we would know them better.’ (FGDs 6, P2, Female, 38 years old with certificate and 3 years of work experience)

Discussions indicated the paucity of parental assistance in the locations. Although some parents value the work of CHWs, many do not want their children to be informed about contraception.

### A lack of respect from teenagers

Participants reported that:

‘Teenagers make them [*CHWs*] a laughingstock in the community by calling them names, that they are denying them *mnandi* [*a good time*] and child grant.’ (FGDs 7, P3, Female, 28 years old with grade 12 and 2 years of work experience)

‘We were told to go and teach our kids and family; however, some are grateful and want to know more and we refer them to the clinic.’ (FGDs 7, P4, Female, 35 years old with grade 12 and 2 years of work experience)

Participants reported that teenagers do not see the value of being aware of the pitfalls of teenage pregnancy, its complications, and consequences. Instead, CHWs are viewed by the youth in their areas as a joke or as people who lack jobs and are using them to earn a living.

## Discussion of findings

All the participants felt that there is a high rate of adolescent pregnancy. The CHWs are of the opinion that the prevalence of teenage pregnancy is increasing rapidly; despite their efforts to educate young people, the rate remains high. The authors of this article are of the opinion that collaborating with CHWs is of paramount importance to achieve better outcomes and successfully tackle the issue of teenage pregnancy. The CHWs identified that the factors that contribute to teenage pregnancy are multifaceted and include a lack of sexual and reproductive health knowledge and awareness, a lack of access to healthcare services, poor communication and miscommunication in churches, a lack of financial support, and a lack of recreational facilities. In addition, poor parenting approaches, peer group pressure, disrupted and/or disorganised households, social media and technology, and alcohol and substance misuse are other problems.

Previous studies have underlined factors that predispose adolescents to pregnancy including culture, early marriage, poor access to modern contraception, low level of education and low socio-economic status.^20,21^ In accordance with the study conducted in South Africa, the study recommended that community-wide initiatives to prevent teenage pregnancy should be supported and strengthened.^11^ According to the findings by Dutta et al., a lack of family support and family customs pushed teenagers towards unwanted and repeated pregnancy.^22^ The Theory of Planned Behaviour, on the other hand, underlines the importance of safe behavioural intentions in addressing teenage pregnancy difficulties.^23^ Parent–child communication on sex, peer interaction, sexuality, explicit material exposure, attitudes, subjective norms and perceived behavioural control is critical to achieving this.^23^

Lowering teenage pregnancy rates is critical to achieving sustainable development goals. Based on the study by Chemutai et al., the rates of teenage pregnancy prevalence vary greatly; in South Africa, they range from 2.3% to 19.2%, whereas in Kenya, they are 31%, 20.4% in Ethiopia, and 31% in Sudan.^1^ This is further supported by the study conducted in Nigeria, where teenage pregnancy grew by 106 per 1000 in 2021, thus indicating that teenage pregnancy affects developing countries.^24^ A study in South Africa indicated that between April 2017 and March 2018, a total of 16 238 children were born to teenage mothers in Limpopo’s state-owned hospitals, with further research showing that Limpopo has the second-highest rate of teenage pregnancy in South Africa.^25^ Participants believe that teenage pregnancy is widespread in their areas. Almost every participant in the focus groups stated a proportion of 80% or higher. However, the study findings went on to reflect those factors that contribute to adolescent pregnancy, as mentioned further in the text. This suggests the critical necessity for substantial measures to be implemented to curb these high teen birth rates.

The participants in this study mentioned that lack of healthcare services, a miscommunication from the church, poverty or a lack of financial support, poor parenting styles, peer pressure, disrupted and/or disorganised families, social media and technology, alcohol and drug abuse, a lack of recreational facilities, and a lack of a sense of belonging and feelings of not being loved are factors that contribute to teenage pregnancy in their rural areas. Several of these factors, including weak family ties, poverty, and a failure to use contraception, have been linked to the prevalence of teenage pregnancy, in accordance with the study conducted in Nigeria.^26^ But the absence of recreational facilities, as well as the issues with social media and technology, were not addressed in their study’s conclusions. This was supported by Kassa et al. who mentioned that living in a rural region, never having been married, not attending school, having a father with minimal education, and parents not talking to their children about sexual and reproductive health concerns are all teenage pregnancy risk factors.^2^

Krugu et al. found that the causes of adolescent pregnancy were broken down into different categories that included: behavioural, environmental, and psychological drivers of sex, sexuality, contraceptive usage and teenage pregnancy.^27^ The girls’ responses appear to have followed certain overlapping patterns. Those who stated they had a good relationship with their parents, for example, reported a desire to further their education and a clear intention to choose safer sex partners to protect their future objectives.^27^ While this is consistent with the study findings, it was not classed as such in the study. The findings also highlighted the difficulties that CHWs confront when it comes to teenage pregnancy, as mentioned further in the text.

Participants discussed the challenges they face on the job, such as a lack of support from parents in the community and a lack of respect from teenagers, when they work with them to implement community awareness campaigns about teenage pregnancy and its effects on teenagers’ health, psychosocial well-being, and economic standing. In the literature, Kumar et al. found that CHWs worked at every health facility and that they all emphasised that adolescent pregnancy was a public health issue.^28^

In a study by Kumar et al. CHWs in Kenya were concerned about adolescent pregnancies and their personal and societal consequences as a public concern.^28^ They also highlighted the challenges of involving men in these communities, as well as the low resources available for basic education on the subject, which contributed to some injustices and their consequences in terms of problems with early and adolescent pregnancy and caregiving. This supports the findings of this study. Furthermore, it has been stated that interaction between the adolescent and the health care facilities is important, and CHWs are well-aware of the obstacles that pregnant adolescents experience at all levels.^28^ The extent literature confirms both this fact and the fact that CHWs were crucial in facilitating the flow of information and access to care between the adolescent and the health care facilities.^12,13,14^ The researcher is of the opinion that they would commonly contact families when an adolescent was pregnant or when a family requested assistance in connecting the facility and resources. They recognise the need for increased openness to take a humanistic and expert approach.

Community health workers can be first-line providers of preventive and intervention services; currently, their work entails bridging gaps between schools, families and healthcare facilities. This is consistent with the findings of Johnson et al., who stated that CHWs are change agents and have been identified as an empowerment method. To make meaningful contributions to society, they require respect from health professionals and the community.^28^ This is an important element to consider when allocating CHWs in communities if health workers want to be recognised as making a difference. Community health workers also lobbied for increased male engagement in Maternal and Child Health (MCH) programmes and improved sex education. The authors are of the view that CHWs were concerned that increasing challenges in the healthcare system, caused by a lack of male participation in MCH services, could undermine the importance of fathers’ roles. The lack of enough trained social workers is making the current problems of performing their work even worse.^26^

### Strengths and limitations

A non-probability purposive sampling relies on the judgement of the researcher and in the end it was a limitation because other members dominated the conversation in the focus groups, which could have potential bias in formulation of themes, because the views of other participants could have been suppressed. As a result, generalisability is not expected in qualitative research. On the other hand, the findings of this study have shared the context being studied, allowing the reader to judge whether the information may be transferred to their situations.

### Implications for clinical practice

The study’s authors suggest a community-based strategy to lower adolescent pregnancy considering the study’s findings. Integrated and improved cooperation with NGOs is necessary so that they can be empowered and taught to cope with community social evils. The Departments of Health, Social Development, and Basic Education must work together with NGO groups to raise awareness and carry out measures to prevent teenage pregnancy. Families, churches, traditional leaders and healers should offer support and assistance on sex education and not avoid the subject. Policy makers need to acknowledge and recognise CHWs’ initiatives and to advocate for adequate material and non-material support such as finances, training and guidelines. Multiple engagements with teenagers should be encouraged to offer health education on teenage prevention, risk of contracting sexually transmitted diseases, and HIV. Churches, communities and traditional leaders should offer guidance on moral regeneration, upholding traditional values and sex education. Platforms for family education need to be initiated so that families are equipped and empowered to address teenage pregnancy challenges. School visits should be scheduled by multiple partners to address the issues of teenage pregnancy. Monitoring and evaluation mechanisms should be put in place in the schools to ensure that learners are being taught about sexual and reproductive health issues. According to the authors’ opinion, CHWs can help address the problem of teenage pregnancy by endorsing programmes and procedures that aim to prevent and control teenage pregnancies.

## Conclusion

The findings of this study highlighted the challenges that CHWs face when offering appropriate teen pregnancy services. There is a lack of trust or a poor relationship between the community and healthcare professionals. As a result, the public has little faith or acceptance in the clinic’s services or system; for example, ‘contraceptives induce infertility’. This is a significant barrier in combating teenage pregnancy; if contraceptives are not acceptable to the community, the only solution and option to combat teenage pregnancy is abstinence.

The CHWs are concerned about the high rate of teenage pregnancy. They felt this was because of inadequate healthcare services, misunderstanding from the church, poverty or lack of financial support, poor parenting styles, peer pressure, disrupted and/or disorganised families, social media and technology, alcohol and drug abuse, a lack of recreational facilities, and a lack of belonging and feelings of not being loved. Community health workers must be trained to help and counsel teens, families, schools, and the community on societal issues such as teen pregnancy, poor parenting practices, and alcohol and substance abuse. Community health workers should have the opportunity to influence policymakers. To inform the public about the effects and consequences of pregnancy in teenagers, more public awareness campaigns must be launched. More research is needed to identify ways to reduce teenage pregnancy.
